# Posttraumatic headache: pain related evoked potentials (PREP) and conditioned pain modulation (CPM) to assess the pain modulatory function

**DOI:** 10.1038/s41598-024-67288-z

**Published:** 2024-07-15

**Authors:** Julia Jessen, Oliver Höffken, Peter Schwenkreis, Martin Tegenthoff, Özüm Simal Özgül, Elena Enax-Krumova

**Affiliations:** Department of Neurology, BG-University Hospital Bergmannsheil, Ruhr-University Bochum, Bürkle-de-la-Camp-Platz 1, 44789 Bochum, Germany

**Keywords:** Posttraumatic headache, Pain-related evoked potentials, Conditioned pain modulation, Endogenous pain inhibition, Central sensitization, Electrical stimulation, Neuroscience, Medical research

## Abstract

Posttraumatic headache (PTH) is common following traumatic brain injury and impacts quality of life. We investigated descending pain modulation as one possible mechanism for PTH and correlated it to clinical measures. Pain-related evoked potentials (PREP) were recorded in 26 PTH-patients and 20 controls after electrical stimulation at the right hand and forehead with concentric surface electrodes. Conditioned pain modulation (CPM) was assessed using painful cutaneous electric stimulation (PCES) on the right hand as test stimulus and immersion of the left hand into 10 °C-cold water bath as conditioning stimulus based on changes in pain intensity and in amplitudes of PCES-evoked potentials. All participants completed questionnaires assessing depression, anxiety, and pain catastrophising. PTH-patients reported significantly higher pain ratings during PREP-recording in both areas despite similar stimulus intensity at pain threshold. N1P1-amplitudes during PREP and CPM-assessment were lower in patients in both areas, but statistically significant only on the hand. Both, PREP-N1-latencies and CPM-effects (based on the N1P1-amplitudes and pain ratings) were similar in both groups. Patients showed significantly higher ratings for anxiety and depression, which did not correlate with the CPM-effect. Our results indicate generalized hyperalgesia for electrical stimuli in both hand and face in PTH. The lacking correlation between pain ratings and EEG parameters indicates different mechanisms of pain perception and nociception.

## Introduction

Posttraumatic headache (PTH) is common after traumatic brain injury (TBI), most frequently after mild TBI^1,2^. It is defined by the International Classification of Headache Disorders (ICHD-3) as a secondary headache, which develops within seven days after trauma, regaining consciousness or recovering the ability to sense and report pain^1^. Persistent PTH lasts per definition for more than 3 months^1^. PTH accounts for 4% of all secondary headache disorders and has high impact on quality of life^3,4^. Its prevalence varies among studies between 10 and 95% of patients with TBI, depending on the severity of trauma and inclusion criteria, but it seems to be still underreported^5–7^. The phenotype of PTH can resemble primary headaches. The most common phenotypes are migraine-like PTH and tension-type-like PTH^1^. Besides the headache characteristics like intensity, localisation and quality, PTH shares additional symptoms with primary headaches^8^, e.g., anxiety, depression, interference of cognitive function, sleep disturbances, or in migraine-like PTH photo- and phonophobia^9,10^. These similarities suggest that they share similar mechanisms, but this seems to be only partly the case^11^.

Overall, the pathomechanisms of PTH are poorly understood, but likely multifactorial. Diffuse axonal injury with remodelling, neurometabolic changes, trigeminal system activation, neuroinflammation and impaired descending pain modulation have been discussed to contribute to the PTH development^11,12^. However, also familiar disposition and psychological factors seem to play a role^11,12^. Despite the high prevalence, little is known about risk factors for developing PTH. History of premorbid headache, female sex, age, accident type and family history of headache have been discussed as contributing factors^11,13^. Evidence-based treatment-options are lacking so far. Current strategies are mostly based on acute or preventive treatments for primary headache disorders, but are not satisfactorily effective^14^. Thus, better understanding of the pathophysiology of pain after TBI can contribute to identify new treatment targets and develop more precise options.

It has been hypothesized that damage to pain-inhibitory pathways leads to hyperexcitability and hyperreactivity of central and peripheral neurons^5^. This, in turn, can lead to generalized allodynia and hyperalgesia which might contribute to the development of posttraumatic headache^5^. We aimed to further investigate these mechanisms using pain-related evoked potentials (PREP) stimulating the hand and forehead to compare results in cephalic and extracephalic areas and to be able to differentiate between generalized hyperexcitability compared to trigeminal activation. Such differences between cephalic and extracephalic regions have been reported in previous studies on primary headache^15,16^.

PREP with concentric surface electrode is a non-invasive examination method measuring the cortical response to painful stimuli and thereby likely the function of the nociceptive system^17^. There are contradicting discussions about whether the concentric surface electrode is selectively capturing the nociceptive or additionally activating large myelinated A-beta-fibres^18,19^. A recent study summarizes the literature, indicating that PREP were altered in peripheral neuropathies of different origin, including small-fibre neuropathy, with prolonged latencies, smaller amplitudes, or missing potentials^20^. Differences in the modulation of PREP compared to somatosensory evoked potentials supports the assumption that PREP correspond to specifically nociceptive activation^21^. In contrast to PREP findings in peripheral neuropathies, PREP results in headache disorders were rather inconsistent, e.g. in migraine patients (see an overview in Bubenzer et al.^20^). PREP latencies were longer and amplitudes were lower in patients with trigeminal neuralgia for trigeminal stimulation compared to the asymptomatic side^22^. Until now, there are no studies on PREP in PTH.

Conditioned pain modulation (CPM) is another method to assess the pain-inhibitory pathways as a net result between descending inhibitory and ascending facilitatory pathways, thus measuring the endogenous pain modulation in humans^23^. This is also known as the “pain inhibits pain”-phenomenon^23^. The method is based on the pain inhibiting effect of a noxious stimulus, called “conditioning stimulus” (CS), on a different noxious stimulus (“test stimulus”, TS) on a remote body site, which is quantified as the CPM-effect, and has been mostly calculated on psychophysical assessment based on changes in pain intensity^24,25^. Recent studies found that patients after TBI exhibit diminished pain inhibitory capacity compared to controls^4,26,27^. Results were mostly based on subjective pain ratings. We have recently introduced a CPM protocol including CPM-effect based on changes in the amplitudes of PCES-evoked potentials as a more objective readout^17^ and applied it to PTH patients for the first time additionally to the PREP-recording.

Based on previous studies we hypothesized higher amplitudes of PREP and a lower CPM-effect in patients compared to healthy controls as result of the increased pain facilitation and decreased pain inhibition. A second aspect focused on the value of CPM and PREP as biomarkers for development and chronification of pain after TBI by relating the study results to clinical data.

## Methods

### Participants

The study was approved by the local ethics committee of Faculty of Medicine, Ruhr-University Bochum, Germany (Reg. Nr. 20-7052, 10-11-2020) and performed according to the latest version of the Declaration of Helsinki. The study including all study details were registered in DRKS in November 2020 (www.drks.de, Reg. Nr. DRKS00023623) prior to study begin. Participants were recruited after informed consent from Department of Neurology, BG University Hospital Bergmannsheil Bochum, Germany between November 2020 and July 2022. All Participants were older than 18 years and had good knowledge of German language.

Patients had to fulfil the criteria for a posttraumatic headache according to the ICHD-3 criteria^1^, with current and average headache intensity (last 4 weeks) of > 2 (NRS 0–10). Exclusion criteria were other severe neurological or psychiatric disorder, a chronic headache disorder before TBI or other pain location with a pain intensity > 5 (NRS 0–10), provided the PTH was reported to be still with stronger pain intensity than other pain locations and the focus of the current treatment. Control subjects were included following the recommendations on inclusion and exclusion criteria for sensory testing, according to them they did not suffer from acute or chronic pain^28^.

### Study design

The initial study was designed with a cross-sectional and longitudinal approach. PTH-patients were first examined at the recruiting point and were invited for a follow-up 3 and 6 months later. The control group had only one examination point. Each session lasted for 2–3 h.

Participants completed questionnaires about headache characteristics, symptoms of depression, anxiety, stress and tendency for pain-catastrophizing (as described below). After neurological examination PREP and CPM was assessed as described below. For the follow-up visits patients completed follow-up questionnaires and underwent the same testing procedure. At the beginning each subject was randomly assigned to group A or B. In group A PREP were recorded after electrical stimulation in the supply area of the right trigeminal nerve region V1 first and afterwards at the dorsum of the right hand (area of the superficial radial nerve), in group B vice versa. CPM was recorded last in both groups. All tests were performed in a low-stimulus room (i.e. well-tempered with no noises, bright lights or interruption), sitting in a relaxed position on a comfortable chair.

### Pain-Related evoked potentials (PREP)

PREP are event-related cortical potentials after painful cutaneous electric stimuli (PCES) and were recorded via EEG with electrodes above Cz referred to linked earlobes (A1 and A2) according to the international 10–20-system^29^. The participants were asked to avoid any movements during recording to improve EEG quality. The nociceptive stimuli were applied using a custom-built concentric surface electrode, in which the anode is an external ring around the cathode^17^. The electrode produces high current density and the depolarisation is limited to superficial skin layers, thus being able to selectively stimulate epidermal nociceptive A-Delta- and C-fibres, without stimulating deeper layered mechanoreceptors (A-Beta-fibres)^17^. The electrode was successively placed at the dorsum of the right hand to stimulate the superficial radial nerve and above the right eyebrow in the trigeminal nerve region V1. The electrode was connected to a stimulator (Digitimer DS7A)).

**Figure 1:**
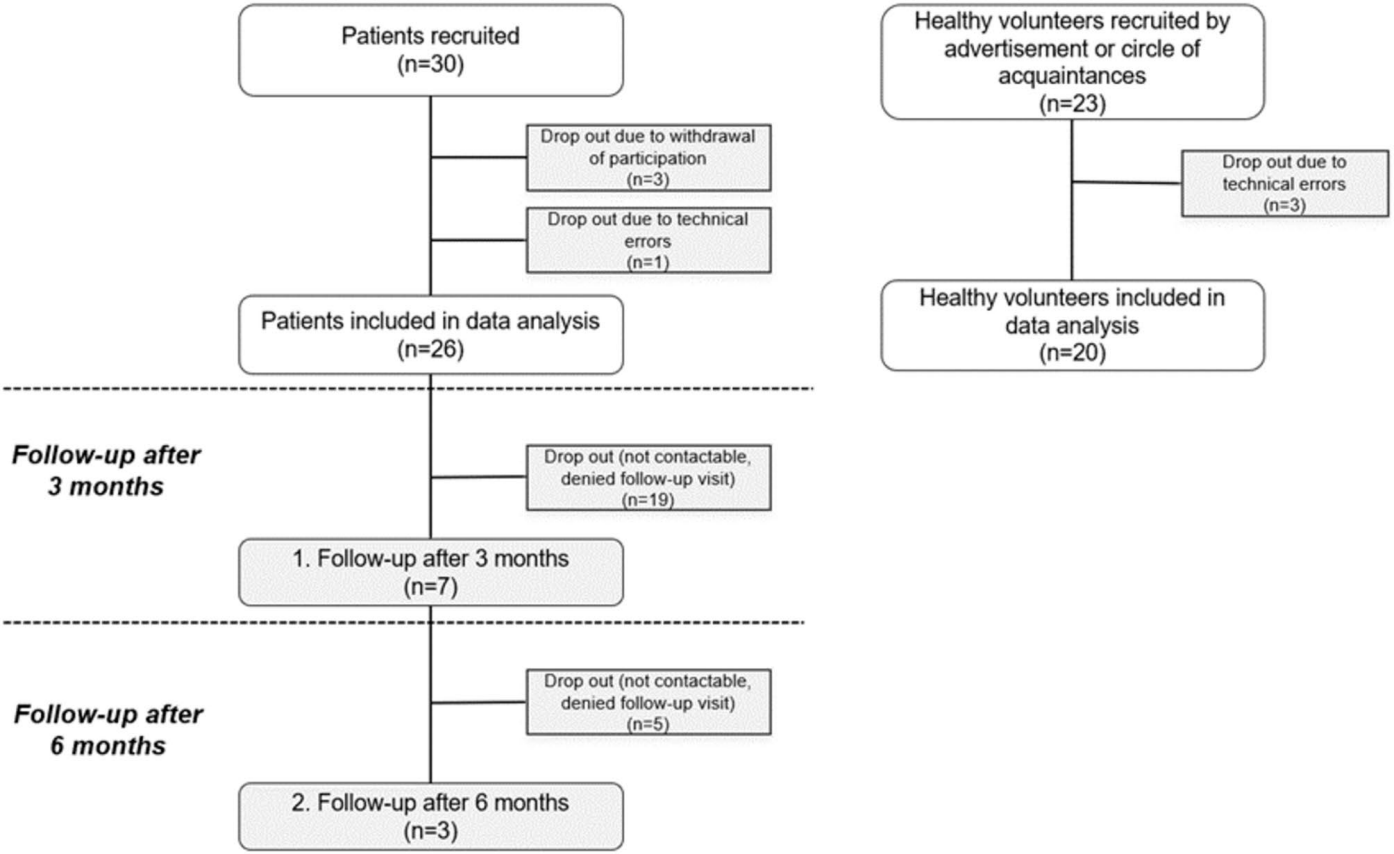
Flow-chart of the patients’ and controls’ recruitment and drop-outs.

**Figure 2:**
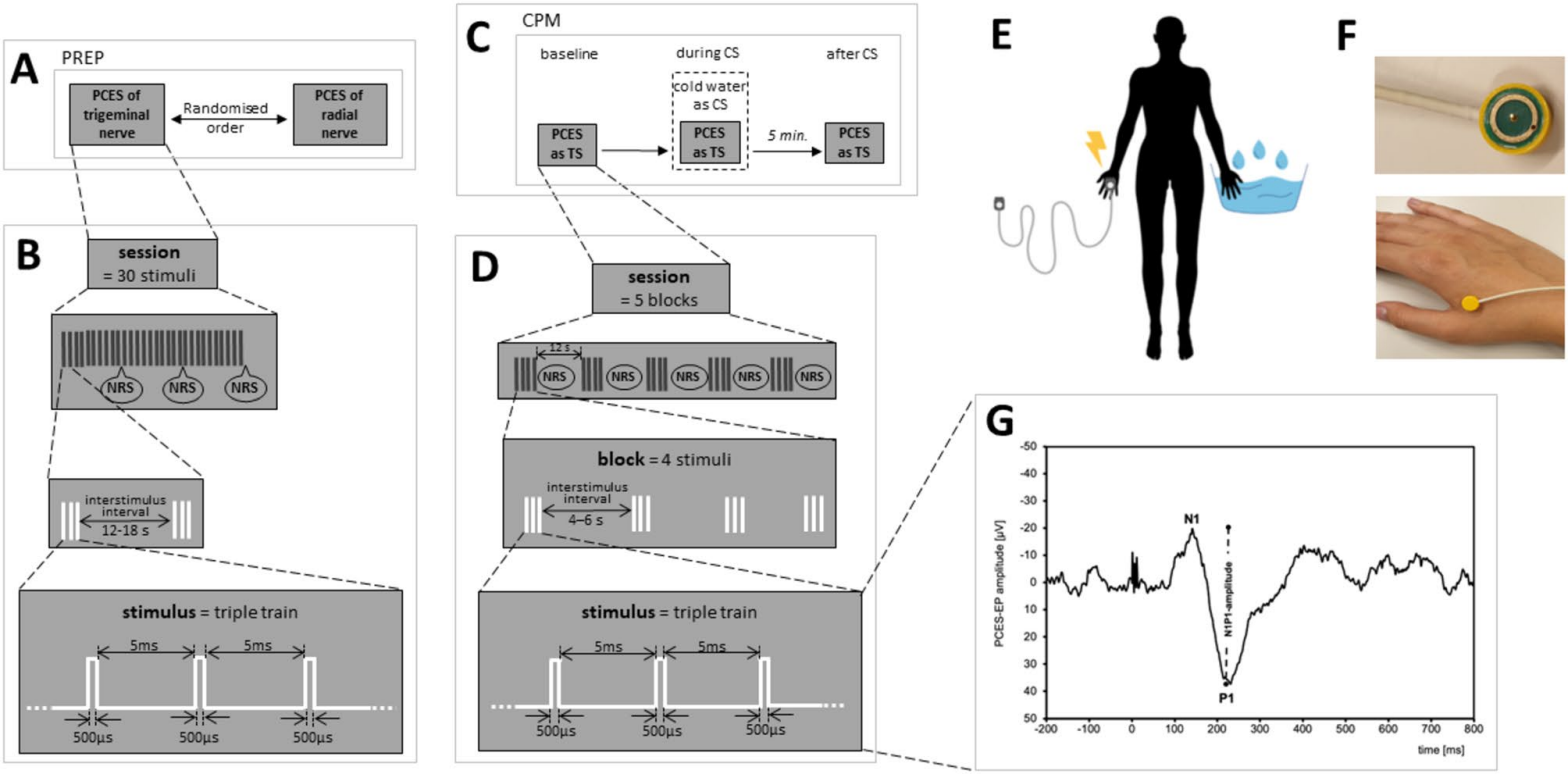
Experimental procedure and stimulation paradigm. (**A**) Timeline of PREP recordings, (**B**) PREP stimulation paradigm, (**C**) timeline of the CPM protocol, (**D**) CPM stimulation paradigm, (**E**) CPM set-up with painful cutaneous electrical stimulation (PCES) of the right hand as the test stimulus and immersion of the left hand in cold water as the conditioning stimulus, (**F**) Picture of the concentric surface electrode and how it is placed on the dorsum of the right hand, (**G**) example of an averaged pain-related evoked potential of one session over Cz according to the international 10–20-EEG-system with N1 as the first negative peak and P1 as the first positive peak. CS: conditioning stimulus, NRS: assessment of the pain intensity on the numerical rating scale (0–100), PCES: painful cutaneous electrical stimulation, PREP: pain-related evoked potentials, TS: test stimulus.

In both areas we first determined the individual detection and pain threshold. The pain threshold was defined as the lowest stimulus intensity at which the subject reports a pinprick-like sensation. The intensity of the electric stimuli was set at the twofold of the pain-threshold, according to most previously published protocols for PREP in other primary headaches and trigeminal neuralgia^30,31^, but also in peripheral neuropathies^17,20,32–34^ to be able to record stable cortical potentials. We applied 30 stimuli, each consisting of a triple pulse (three successive monopolar square waves with a 500 μs duration and an inter-wave interval of 5 ms) with an interstimulus-interval of 12–18 s. After each 10 stimuli the participant was asked to rate the painfulness of the last stimulus on a numeric rating scale (NRS) from 0 to 100 (0 = no pain, 100 = maximum pain imaginable) (Fig. 2A, B and F). The duration of the examination session for PREP recordings was approx. 20 min.

The EEG was analysed by examining the time frame 200 ms before to 800 ms after stimulus onset and editing with a baseline correction and a notch filter (Brain Amp, Brain Products, Gilching, Germany; bandwidth: 1 Hz to 1 kHz; sampling rate: 5 kHz). At each measure we rejected the first stimulus to avoid movement artifacts due to the arousal reaction and manually removed any other artifacts due to blinking or technical error, as previously established^16,17,35^. After averaging the potentials, N1-latencies and N1P1-amplitudes of the potentials were determined by one examiner (JJ under the direct supervision of ÖÖ).

### Conditioned pain modulation (CPM)

CPM was assessed directly after PREP with the same EEG set-up.

We used PCES on the dorsum of the right hand as TS and the immersion of the left hand into a 10°C cold water bath as CS, as previously introduced^35^. The TS was set at the current intensity the subject rated as 60 on the NRS 0–100. The assessment was divided in 3 sessions. For each session the TS was applied 20 times. Each 4 stimuli built a block with 4–6 s interstimulus interval. The interval between the blocks was 12 s. After each block the subject was asked to rate the perceived pain on the NRS. In the first session the TS was applied alone, as described (baseline). In the second session the participant”s left hand was submersed to the wrist in the cold-water bath 20 s before the PCES started. The subject was asked to rate the perceived left (CS) and right (TS) hands pain after each 4 stimuli separately (during). The application time of the water was 2 min. After 5 min break the protocol was repeated without CS (after) to assess any longer lasting effects of the CS (Fig. 2C–E). The duration of the CPM assessment was approx. 15 min.

**Figure 3:**
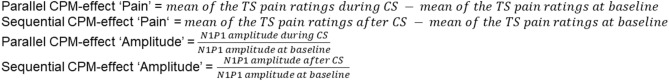
Calculation of the parallel and sequential CPM-effects based on the subjective pain ratings and the N1P1-amplitudes in the EEG as an objective readout.

The mean pain rating of each session (baseline, during, after) was defined as the mean of the 5 pain ratings after each block. For the analysis of CPM we calculated the CPM-effects, as shown in Fig. 3. The parallel and sequential CPM-effect “pain” referred to the pain ratings and was calculated as the difference between the mean pain ratings; accordingly, every value < 0 was suggested to represent an efficient pain inhibition in both “Pain”-CPM-effects. The parallel and sequential CPM-effect “amplitude “ was set as the quotient between the means of the N1P1-amplitudes; accordingly every value < 1 was suggested to represent an efficient pain inhibition. Lower values indicated greater endogenous pain inhibition^35,36^.

### Questionnaires

We used following questionnaires (German versions) to assess headache characteristics in patients and psychological variables in both groups:

*German Pain-Questionnaire (Deutscher Schmerzfragebogen, DSF).* The DSF examines pain characteristics, as well as psychological and social factors.

*Kiel Headache Questionnaire (Kieler Kopfschmerzfragebogen, KKF).* KKF is a 26-item questionnaire used to assign the headache to one of the primary headache entities migraine, episodic tension-type headache or chronic tension-type headache.

We used the DSF and the KKF to characterize the headache in patients and categorize it finally in mostly tension-type-like or mostly migraine-like type, as can be seen in Table 1.

**Table 1:** Clinical and demographic data of patients with PTH compared to healthy controls (where appropriate).

	Patients with PTH (*n* = 26)	Controls (*n* = 20)	*p*-value
Age (years, mean ± SD)	41.9 (± 11.1)	40.7 (± 15.5)	0.757
Sex (female, *n* (%))	5 (19.2%)	7 (35.0%)	0.250
Questionnaires			
PSQ score (mean ± SD)	4.5 (± 1.6)	4.5 (± 1.4)	0.925
PCS score (mean ± SD) PCS score > 30 (*n* (%))	25.2 (± 14.2) 9 (34.6%)	12.6 (± 8.2) –	< 0.001***
HADS-A score > 7 (*n* (%))	20 (76.9%)	1 (5.0%)	< 0.001***
HADS-D score > 7 (*n* (%))	18 (69.2%)	2 (10.0%)	< 0.001***
Clinical characteristics			
Duration of headache symptoms (weeks, mean ± SD)	92 (± 239)	–	
Average headache intensity in last 4 weeks (NRS (0–10), mean ± SD)	6 (± 1.8)	–	
Maximum headache intensity in last 4 weeks (NRS (0–10), mean ± SD)	8.4 (± 1.7)	–	
Current headache intensity (NRS (0–10), mean ± SD)	3.9 (± 2.4)	–	
Pain intensity (high, *n* (%))#	21 (80.8%)	–	
Disability (high, *n* (%))#	22 (84.6%)	–	
Headache attack frequency			
Constant pain (*n* (%))	14 (53.8%)	–	
Several times daily (*n* (%))	6 (23.1%)	–	
Once daily (*n* (%))	1 (3.8%)	–	
Several times weekly (*n* (%))	3 (11.5%)	–	
Several times monthly (*n* (%))	2 (7.7%)	–	
Description of headache			
Pressure, tight or squeezing (*n* (%))	21 (80.8%)	–	
Pounding or throbbing (*n* (%))	12 (46.2%)	–	
Stabbing or pulling (*n* (%))	22 (84.6%)	–	
Cause of injury			
Fall of height (*n* (%))	9 (34.6%)	–	
Traffic accident (*n* (%))	8 (30.8%)	–	
Hit to the head (*n* (%))	4 (15.4%)	–	
Hit and fall on the head (*n* (%))	3 (11.5%)		
Other (*n* (%))	2 (7.7%)	–	
Severity of TBI			
Mild (*n* (%))	15 (57.7%)	–	
Moderate (*n* (%))	4 (15.4%)	–	
Severe (*n* (%))	7 (26.9%)	–	
Loss of consciousness			
Yes (*n* (%))	16 (61.5%)	–	
No (*n* (%))	10 (38.5%)	–	
Pain medication			
Regular (*n* (%))	10 (38.5%)	–	
On-demand (*n* (%))	10 (38.5%)	–	
None (*n* (%))	6 (23.1%)	–	
Headache type			
Mostly tension-type-like headache (*n* (%))	12 (46.2%)	–	
Mostly migraine-like headache (*n* (%))	10 (38.5%)	–	
Both/not specified (*n* (%))	4 (15.4%)	–	
Additional symptoms			
Concentration problems	21 (80.7%)	–	
Increased irritability	12 (46.2%)	–	
Dizziness	11 (42.3%)	–	
Anxiety	11 (42.3%)	–	
Sleep disturbance	10 (38.5%)	–	
Impaired vision	9 (34.6%)	–	
Depressiveness	8 (30.8%)	–	
Other	6 (23.1%)	–	

*HADS-A* Hospital anxiety and depression scale-anxiety, *HADS-D* Hospital anxiety and depression scale-depression, *PCS* Pain catastrophizing questionnaire, *PSQ* Pain sensitivity questionnaire, *TBI* Traumatic brain injury, * significant *p*-value (* = *p* < 0.05, ** = *p* < 0.01, *** = *p* < 0.001), # according to v. Korff^40^.

*Pain Sensitivity Questionnaire (PSQ).* The PSQ was used to evaluate the individual self-assessed pain sensitivity. It contains 17 questions about everyday life painful events, rated from 0 to 10 on NRS (0 = not painful, 10 = the most imaginable pain)^37^.

*Pain Catastrophizing Scale (PCS).* The tendency to catastrophize pain, as well as the relation of negative expectations, emotional distress and pain maintenance was assessed by this scale. 13 statements describe cognitions and situations during pain states and can be answered from 0 = ”not at all” to 4 = ”all the time”. Higher PCS-scores indicate higher pain catastrophizing (catastrophizers: score > 30)^38^.

*Hospital Anxiety and Depression Scale (HADS).* HADS is a 14-item questionnaire that evaluates anxiety and depression symptoms (abnormal: score > 7 separately for the depression and anxiety subscales)^39^.

### Statistical analysis

Our sample size estimation was based on data from previous studies on patients with headache disorders (episodic migraine, *n* = 16, or medication overuse headache, *n* = 14), where PREP amplitudes were significantly higher compared to healthy subjects (*n* = 15)^16^. Based on these data^16^ with an expected difference of the PREP amplitude of at least 9 µV (4.5 µV standard deviation), a desired *p*-value < 0.01 and a power of at least 90, a sample size of at least 16 patients and control subjects each was calculated as sufficient. We planned to recruit 30 patients and 20 healthy controls.

The statistical analysis was conducted with IBM SPSS software (version 28.0). The threshold for statistical significance was set at *p* < 0.05. First, we examined if the data were normally distributed using the Shapiro–Wilk-Test. For the descriptive statistics we calculated means and standard deviations for continuous variables and frequencies for categorical variables. To compare parameters between both study groups (patients vs. control) we used the unpaired t-test. Violations in variance homogeneity were balanced using the Welch-test instead. Additionally, we conducted 2 × 3 mixed ANOVA to analyse differences in amplitude and pain rating during CPM procedure with 3 assessment time points (baseline, during CS, after CS), again comparing the two study groups (patients vs. control). Greenhouse–Geisser adjustment was used to correct for violations of sphericity. Pearson correlation analysis examined associations between variables (DT, PT, age, amplitudes, pain ratings, results from the questionnaires, PTH duration; see results).

### Ethical approval and patient consent

The study was approved by the local ethics committee of Faculty of Medicine, Ruhr-University Bochum, Germany (Reg. Nr. 20-7052, 10-11-2020) and performed according to the latest version of the Declaration of Helsinki. Participants were included into the study after written informed consent.

## Results

### Participants

We included 26 PTH-patients and 20 age-matched healthy control subjects. Only 7 patients showed up for the follow-up visit after 3 months and 3 of them participated at the second follow-up after 6 months (Fig. 1). Because of the high dropout rate and the small sample size at follow-up, we here only report data from baseline. Our data was normally distributed. Clinical and demographic data of both groups are shown in Table 1^40^. Twelve patients reported that their headache was accompanied by pain in the neck areas, and two reported that it radiated also in the back. Due to our recruitment in our clinic as a centre for workplace accidents, we had 5 patients with other pain locations, mostly due to polytrauma with other fractures additionally to the TBI. Patients and controls did not differ significantly in sex or age. PTH-patients had an average headache intensity of 6 (± 1.8) (NRS 0–10) with a mean duration of 92 (± 239) weeks. Patients were included with any TBI severity. 6 patients took different antidepressants as co-analgesics.

### PREP

The mean pain ratings during electrical stimulation in both face and hand were significantly higher in patients than in controls, despite similar stimulation intensities (Table 2). In contrast, the PREP-amplitudes were lower in patients than in controls, though the level of significance was only reached during hand stimulation (*p* = 0.04). The N1-latencies as well as the perception and pain thresholds showed no significant differences between both groups.

**Table 2:** Parameters of the recording of pain-related evoked potentials (PREP).

	Patients with PTH	Controls	*p*-value
DT forehead (mA, mean ± SD)	0.5 ± 0.2	0.4 ± 0.2	0.248
PT forehead (mA, mean ± SD)	0.6 ± 0.3	0.6 ± 0.3	0.813
DT hand (mA, mean ± SD)	0.9 ± 0.3	0.7 ± 0.3	0.102
PT hand (mA, mean ± SD)	1 ± 0.4	1 ± 0.5	0.854
Pain rating forehead (NRS, mean ± SD)	41.8 ± 26.1	19.6 ± 18	0.002**
N1-latency forehead (ms, mean ± SD)	138.2 ± 17.6	136.9 ± 22.7	0.834
N1P1-amplitude forehead (mV, mean ± SD)	24.7 ± 12.8	28.5 ± 13.1	0.334
Pain rating hand (NRS, mean ± SD)	37.7 ± 26.5	20.5 ± 17.5	0.011*
N1-latency hand (ms, mean ± SD)	154.6 ± 18.3	148.6 ± 25.5	0.357
N1P1-amplitude hand (mV, mean ± SD)	22.6 ± 11.6	30.6 ± 13.9	0.040*

*DT* Detection threshold, *PT* Pain threshold, *Significant *p*-value (* = *p* < 0.05, ** = *p* < 0.01, *** = *p* < 0.001).

Significant values are in bold.

### CPM

**Figure 4:**
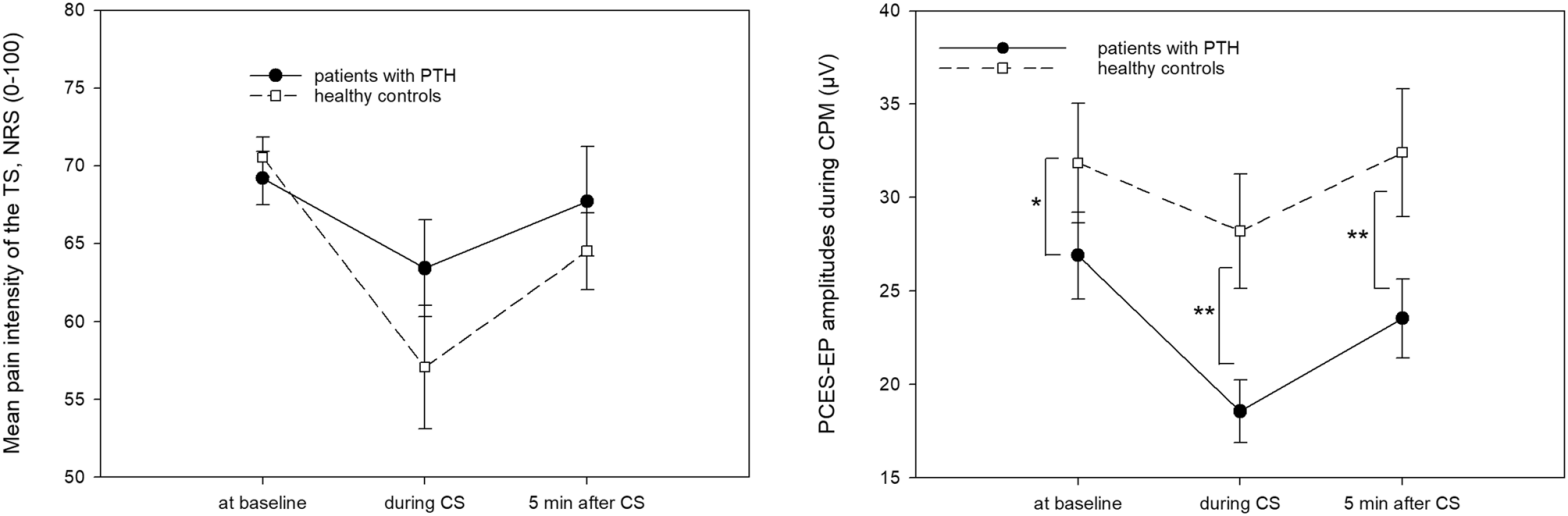
Results of the CPM procedure. (**A**) Mean pain intensity of the test stimulus (TS) in patients and controls, (**B**) Mean N1P1-amplitude of evoked potentials (EP) after painful cutaneus electrical stimulation (PCES). Bars representing standard error of the mean. *significant effect.

Comparing the two groups (Table 3), patients reported a pain rating of 60 on a NRS 0–100 after electrical stimulation with significantly lower current intensity than controls (*p* = 0.041). The N1P1-amplitudes were significantly lower in patients than in controls in all CPM measurements (Table 3, Fig. 4). The CPM-effects varied interindividually both based on the changes in pain intensity and N1P1-amplitudes. Mixed ANOVA revealed a significant CPM-effect “Pain” and “Amplitude” in both groups, but there was no statistically significant interaction between amplitudes and group or pain ratings and group (Table 4).

**Table 3:** CPM-parameters.

	Patients with PTH	Controls	*p*-value
NRS 60 rated current intensity (mA, mean ± SD)	7.3 ± 5.3	15.1 ± 15.4	0.041*
Pain rating baseline (NRS, mean ± SD)	67.3 ± 9.6	69.5 ± 8.2	0.419
Pain rating during CS (NRS, mean ± SD)	60.9 ± 14.4	57.8 ± 18.3	0.525
Pain rating of CS (NRS, mean ± SD)	58.0 ± 25.3	50.8 ± 22.8	0.323
Pain rating after CS (NRS, mean ± SD)	64.2 ± 16.9	63.1 ± 11.8	0.793
N1P1-amplitude baseline (mV, mean ± SD)	27.1 ± 10.9	36.5 ± 13.6	0.013*
N1P1-amplitude during CS (mV, mean ± SD)	22.0 ± 10.0	34.8 ± 20.5	0.008**
N1P1-amplitude after CS (mV, mean ± SD)	24.2 ± 9.8	34.5 ± 13.3	0.004**
Early CPM-effect “pain”	− 6.4 ± 10.4	− 11.7 ± 16.4	0.190
Late CPM-effect “pain”	− 3.1 ± 11.7	− 6.6 ± 10	0.305
Early CPM-effect “amplitude”	0.9 ± 0.4	0.9 ± 0.3	0.465
Late CPM-effect “amplitude”	0.9 ± 0.2	1.0 ± 0.2	0.476

*CS* Conditioning stimulus (cold water), *NRS* Numeric rating scale (0–100), * significant *p*-value (* = *p* < 0.05, ** = *p* < 0.01, *** = *p* < 0.001).

Significant values are in bold.

**Table 4:** ANOVA for CPM parameters.

	Main effect			Main effect for group			Interaction *group		
	Greenhouse–Geisser	Significance	Partial eta-square	Greenhouse–Geisser	Significance	Partial eta-square	Greenhouse–Geisser	Significance	Partial eta-square
Evoked pain	F_1,67;73,62_ = 14.751	*p* < 0.001***	0.251	F_1;44_ = 0.037	*p* < 0.001***	0.001	F_1,67;73,62_ = 1.282	*p* = 0.280	0.028
N1P1-amplitude	F_1,46;64,04_ = 2,974	*p* = 0.074	0.063	F_1;44_ = 9.351	*p* = 0.004**	0.175	F_1,46;64,04_ = 0.793	*p* = 0.420	0.018

*Significant *p*-value (* = *p* < 0.05, ** = p < 0.01, *** = p < 0.001).

Significant values are in bold.

The parallel and sequential CPM-effect “Pain” (*r* = 0.745, *p* < 0.001) and “Amplitude” (*r* = 0.537, *p* < 0.001) correlated significantly. However, there was no significant correlation between CPM-effects “Pain” and the CPM-effects “Amplitude”.

### Questionnaires

There was no difference in the self-reported pain sensitivity between patients and controls assessed by PSQ. The tendency to catastrophize pain assessed by PCS was significantly higher in patients than in controls (*p* < 0.001). HADS revealed pathological scores in patients for depressive (in 77%) or anxiety symptoms (in 70%) and was significantly higher than in controls.

### Correlations

Pearson correlation analysis showed a moderate positive correlation between the detection and pain thresholds at the hand with the detection (*r* = 0.502, *p* < 0.001) and pain (*r* = 0.620, *p* < 0.001) thresholds at the forehead. Age correlated negatively with the N1P1-amplitudes of PREP at both stimulation sites (forehead: r = − 0.591, *p* < 0.001; hand: r = − 0.369, *p* = 0.012), meaning older participants had lower N1P1-amplitudes. We found a moderate negative correlation between the pain rating of the CS and the N1P1-amplitude during the application of the CS (r = − 0.368, *p* = 0.012). Hence, higher CS pain intensities were associated with lower N1P1-amplitudes during CS application. Higher average headache intensity was moderately associated with a higher PCS score (r = 0.424, *p* = 0.031) and anxiety score in HADS (r = 0.463, *p* = 0.017). The anxiety scores in HADS were also moderately negative correlated with PREP-amplitude of the trigeminal stimulation (r = − 0.303, *p* = 0.041) and the amplitudes in the CPM procedure (before CS: r = − 0.325, *p* = 0.028; during CS: r = − 0.355, *p* = 0.015; after CS: r = − 0.362, *p* = 0.014). Thus, higher anxiety scores came along with lower amplitudes. PCS scores correlated moderately positive with the pain rating after PCES (forehead: r = 0.292, *p* = 0.049; hand: r = 0.366, *p* = 0.012), participants with higher tendency to catastrophize reported higher pain sensation. There was no correlation to the duration of PTH.

## Discussion

The aim of the study was to investigate the pain modulatory function in patients with PTH as a possible contributing mechanism, based on a novel recently established testing paradigm CPM^35,41,42^ including electrophysiological parameters after electrical stimulation as an objective readout. Additionally, we recorded PREP as previous studies on primary headaches and trigeminal neuralgia indicated that increased PREP amplitudes might be useful as a marker for central sensitisation^16,43,44^.

To our knowledge, this is the first study recording PREP in patients with PTH. According to our results, PCES evoked stronger pain in patients than in controls despite equal stimulation intensities. However, the N1P1-amplitudes of PREP were lower in patients after hand stimulation. CPM revealed higher pain sensitivity to PCES and a reduced N1P1-amplitude in patients already at baseline, but no group differences in the CPM-effect.

### Reduced N1P1-amplitudes in PTH

Contrary to our hypothesis, both the N1P1-amplitudes after PCES at baseline, during and after CPM assessment and PREP after stimulation of the hand were significantly lower in patients compared to controls. The lower amplitudes after PCES during CPM assessment could be explained by the lower stimulus intensity at which patients reported a pain rating of NRS 60, which could be interpreted as a sign of sensitization, but the stimulation intensities did not differ in the PREP measurement. Another possible explanation for the reduced amplitudes in PTH patients could be that the endogenous pain modulation is constantly in active state due to the persistent spontaneous pain in PTH and the CS during experimental CPM assessment cannot further activate the descending pain inhibitory pathways.

Our patients also had higher burden due to depressive symptoms, which might possibly influence the PREP response. Unfortunately, there are no studies on PREP or CPM in depressive disorders. Loudness dependence of auditory evoked potentials (LDAEP) has been discussed to reflect central serotonergic activity^45^, which is also important for endogenous pain inhibition^36,46^. However, increased LDAEP in depression was found by one study^47^, but not in others^48,49^. Further studies comparing both methods might elucidate any effects on depression on PREP results.

In contrast to our findings, higher PREP amplitudes were reported in medication overuse headache (MOH) with normalisation after withdrawal when using the same concentric surface electrode and stimulation sites for PREP testing as in our study^16^. Our patients were also under treatment with different pain medication, 10 patients taking analgetic medication daily and thereby meeting the criteria for MOH. The contradicting results lead to the assumption that the underlying mechanisms in PTH are others than in MOH arising from primary headaches.

### Increased pain sensitivity after PCES in PTH

In our study, the patients reported higher pain intensities during PREP recording despite similar stimulation intensities. Likewise, patients needed lower stimulation intensities to evoke a pain of 60 NRS during CPM. The higher pain sensitivity might be explained by an arousal due to their headache. Alternatively, trauma might have led to hyperexcitability of the central nociceptive system e.g. on a spinothalamic/thalamocortical level or in the brainstem which contributes to the chronic pain. On the other hand, the pain ratings of the CS did not differ significantly between groups. Additionally, 6 patients were under the influence of antidepressants as part of the analgetic treatment. A medication effect on the descending inhibitory system cannot be excluded, although visually analysing the data, there was no difference to patients without antidepressive treatment, but statistical analysis was not possible due to the small subgroups.

### Impaired CPM as a potential mechanism in PTH

There was no interaction either for amplitudes or pain ratings and group during CPM, in contrast to a longitudinal study in PTH-patients (with the same CS on the right and pressure pain as TS on the left arm), where an interaction for the group was reported^26^. This discrepancy might result from the timepoint of recruiting, as Naugle et al. enrolled patients only at an early stage after trauma, while we had no fixed timepoint, with the earliest timepoint being 10 weeks post trauma. Findings at earlier stages could be explained by direct affection of the peripheral nerves. Therefore, Bouferguene et al.^50^ recommends only enrolling patients > 12 months after trauma to be able to assess long lasting mechanisms. However, our findings did not correlate with headache duration. Effects during CPM procedure could be questioned as being habituation effects, however in a previous study assessing the same testing paradigm we demonstrated that the CPM effect is larger than habituation effects after repeated PCES^41^. Interestingly, the CPM effect assessed by our paradigm also seem not to result from a mere distraction from the primary pain stimulus^51^. We used the established CPM paradigm with application of the test and CS on the hand. To date, only a few studies conducted a CPM protocol stimulating the head/face region. One study by Williams et al. assessed CPM in migraine patients with electro-cutaneous stimulation of the left supraorbital branch as TS and forearm ischaemia as CS with pain rating as subjective readout^52^. They found a significant inhibition in the pain ratings in migraine patients and controls, but no differences between the groups^52^. Another study (Exposto et al., 2021) evaluated CPM in tension-type headache with pressure pain to temple area as TS and cold water immersion of the foot as CS and also found no significant differences between the groups^15^. However, it cannot be excluded that results in PTH might differ in case of stimulus application in the trigeminal area.

It remains unclear whether impaired CPM capacity is a risk factor for pain chronification or if pain leads to impaired CPM capacity. Studies on prediction of postoperative pain indicate that lower pain modulation capabilities in patients without chronic pain before surgery can lead to higher pain post-surgery^53^. Studies on pain perception in primary headaches, using other CPM protocols and nociceptive blink reflex among others, found evidence that impaired CPM in trigeminal area, suggesting deficient nociceptive inhibition could contribute to the development of headache^52,54^.

### Central sensitisation as a potential mechanism in PTH

Direct damage to the cranial nerves is suspected to lead to neurogenic inflammation and hyperreactivity of peripheral nociceptors. With our methods, no (possibly subclinical) lesion in the trigeminal area could be detected in the standardized testing areas. However, 7 patients reported circumscribed hypesthesia in other facial areas. Overall, the higher sensitivity to pain in headache patients did not correlate with the potential amplitudes elicited by PCES. Analogous to animal experiments, the hyperalgesia in our patients is not limited to the painful body region, but can be found in extracranial regions^55^. Interestingly, the hyperalgesia in extracranial regions was not found in all animal studies, implicating that there are further underlying factors^55^. The presence of extracephalic hyperalgesia implicates affection of central pathways, which was previously interpreted as feature of central neuropathic pain^8^. Diminished pain modulatory capacity has been discussed in previous studies as a contributing mechanism to chronic headache after TBI^27^, which might be due to damaged ascending spinothalamic/thalamocortical tracts or due to damage in the brainstem leading to hyperexcitability^56,57^. This could include direct tissue damage, but also changes in transmitter release or cerebral blood flow^5^. Thus, impaired afferent cranial input seems to be a prerequisite in addition to the generally impaired endogenous inhibition with hypersensitivity in the hand region, as the pain is located only in the cephalic region and not generalized. With regard to our study, this could mean that sensitization and impaired descending inhibition can be found in the facial region of patients in addition to altered peripheral nociceptive input. Whereas, in the hand area the active descending top-down inhibition might contribute to the reduced the amplitudes only extracranial. Interestingly, a recent study in animal model showed, that calcitonin-gene related peptides could block the allodynia in acute stage after TBI and prevent the loss of diffuse noxious inhibitory controls in mice^58^.

### Correlation to clinical parameters

Further, we could not confirm our hypothesis that higher average headache intensities are associated with greater impairment in pain perception in patients. This was already previously found in other studies on CPM in headache patients^52,54,57,59^. We could only show that high average pain intensity goes along with higher burden of psychological symptoms. We found high scores of pain catastrophizing, anxiety and depressive symptoms in PTH-patients, similar to previous studies^27^. The correlation between these symptoms and the PTH intensity was also previously described^27^. It remains unclear whether coexisting psychological factors lead to higher headache intensities or higher headache intensities after trauma leads to psychological impairments, but most likely they reinforce one another. The lack of correlation between CPM effects based on subjective pain ratings and N1P1-amplitudes indicates different mechanisms involved in brain activity elicited by nociceptive stimuli and subjective pain perception, as it has been previously discussed in another context^60,61^. Research on this topic indicates the pain perception being divided in sensory-discriminative and affective-motivational part and involving different brain regions^61^. Although patients showed higher pain ratings in our study, their self-assessed sensitivity to pain did not differ in the PSQ. Possibly the patients based their assessments on their experiences before the trauma or they have missing awareness of their pain hypersensitivity.

### Limitations and future directions

Due to differing injury mechanisms the patient collective is heterogenous. Some patients were under pain medication during the study measurements, however only patients who still reported a headache intensity of NRS > 2 (0–10) were included. Moreover, we recruited PTH-patients at any time after TBI. The variance of the time point was very high and we cannot make a statement about the further course of the headache over time. Due to the focus of our clinic as a centre for workplace accidents, more male than female patients (e.g. more male workers in construction sites) were recruited. This could be a bias, because female sex is a discussed risk factor for PTH development and could underly different neurophysiological responses. Moreover, we had 5 patients with other pain location (with intensity up to NRS 5 according to the inclusion criteria), mostly due to polytrauma, but they were only included if the headache was more intense and the focus of the treatment. Psychological factors or medication overuse could be overlaying and contributing initial mechanisms. In our study we did not include a control group of patients after TBI without headache. In further studies it would be interesting to analyse their pain modulatory capacity similarly and at a defined timepoint after trauma to possibly find risk factors for PTH development. Our relatively small sample size allowed us indeed to see statistical relevant effects but we could not analyse patient subgroups, for example split by phenotype. Further, due to recruitment restraints we could not include enough patients with acute PTH to make assumptions on mechanisms contributing to chronification. Our CPM protocol used 10 °C cold water as the CS, but there are differences in the individual temperature sensitivity, so possibly the inhibitory effect might have not been sufficient in individuals that rated the water bath as less painful (5 patients and 3 controls rated the CS ≤ 20 NRS). Since not every TBI patient develops a headache, especially longitudinal studies are needed to analyse the disease progress and find modulating factors. Future studies should further evaluate the role of PREP as a method to analyse effect of targeted pain-modulating medication in PTH or other pain states^21^.

## Conclusion

Our findings show signs of hyperalgesia in PTH-patients in cephalic and extracephalic regions. Despite lower amplitudes in patients, they reported subjectively higher pain sensation in painful (face) and remote (hand) site. The lower amplitudes can either result from existing central lesions in the examined pathway or from inhibitory top-down mechanisms, as we only measure the final cortical response. The lack of correlation between subjective pain ratings and objective EEG parameters could indicate different pathways of pain perception and nociception. The results are not sufficient to be able to use the methods as biomarker for pain development after TBI yet. Therefore, we need more (longitudinal) studies with bigger cohorts. Management and therapy after TBI need to be improved, because it is a severely debilitating disease that often affects young people and current treatment options are not specific and unsatisfactorily effective^14^.

### Supplementary Information

Supplementary Information.

## Data Availability

The data that support the findings of this study are available on request from the corresponding author. The data are not publicly available due to privacy or ethical restrictions.
