# Supplementary material for: Posttraumatic headache: pain related evoked potentials (PREP) and conditioned pain modulation (CPM) to assess the pain modulatory function
Source: Sci Rep. 2024 Jul 15;14:16306. doi: 10.1038/s41598-024-67288-z (PMC11251016; doi:10.1038/s41598-024-67288-z)
Supplement: Supplementary file 1 — Supplementary Information. [file 41598_2024_67288_MOESM1_ESM.pdf]

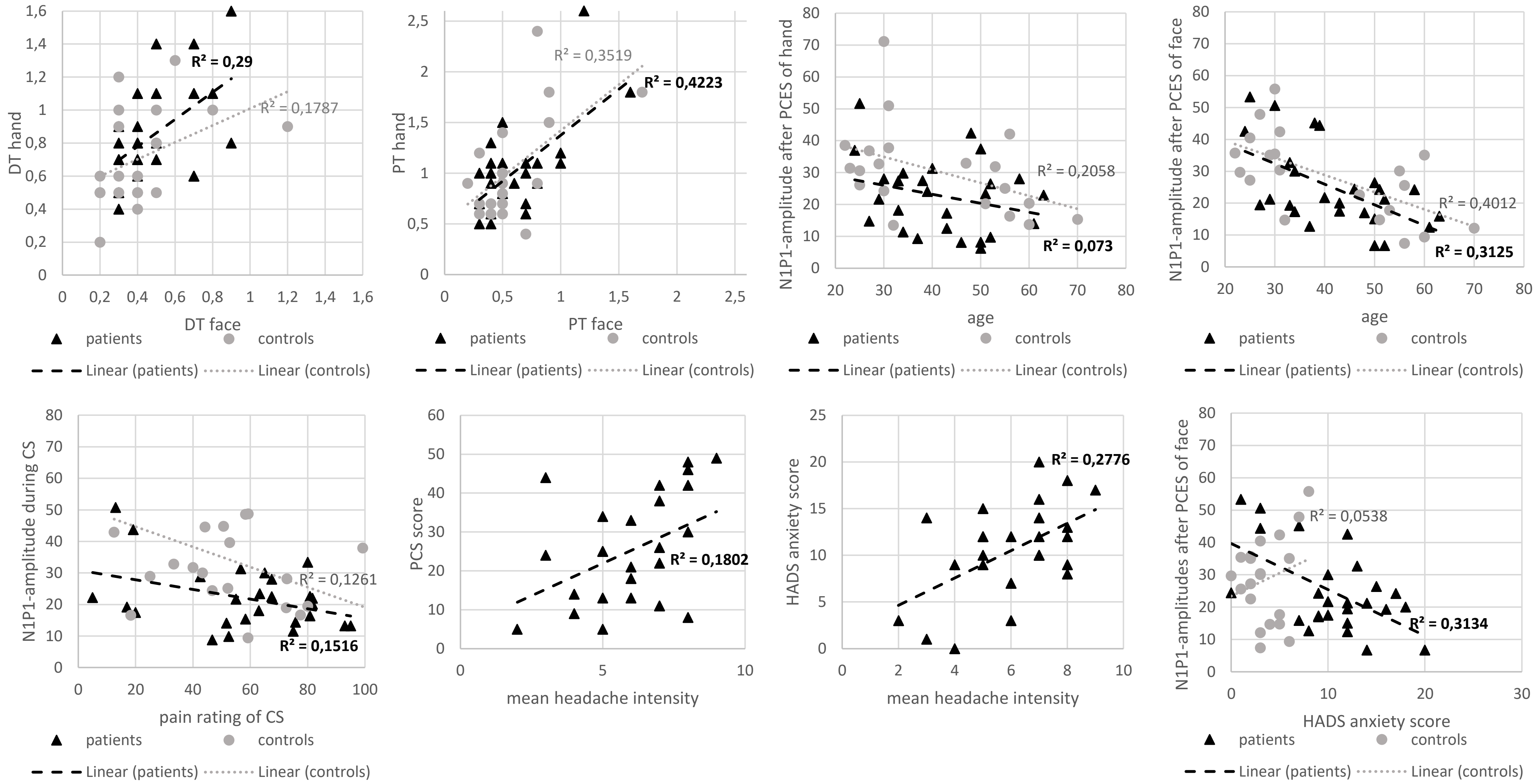
DT hand
DT face
R² = 0,29
R² = 0,1787
PT hand
PT face
R² = 0,3519
R² = 0,4223
N1P1-amplitude after PCES of hand
age
R² = 0,2058
R² = 0,073
N1P1-amplitude after PCES of face
age
R² = 0,4012
R² = 0,3125
N1P1-amplitude during CS
pain rating of CS
R² = 0,1261
R² = 0,1516
PCS score
mean headache intensity
R² = 0,1802
HADS anxiety score
mean headache intensity
R² = 0,2776
N1P1-amplitudes after PCES of face
HADS anxiety score
R² = 0,0538
R² = 0,3134
patients
controls
Linear (patients)
Linear (controls)

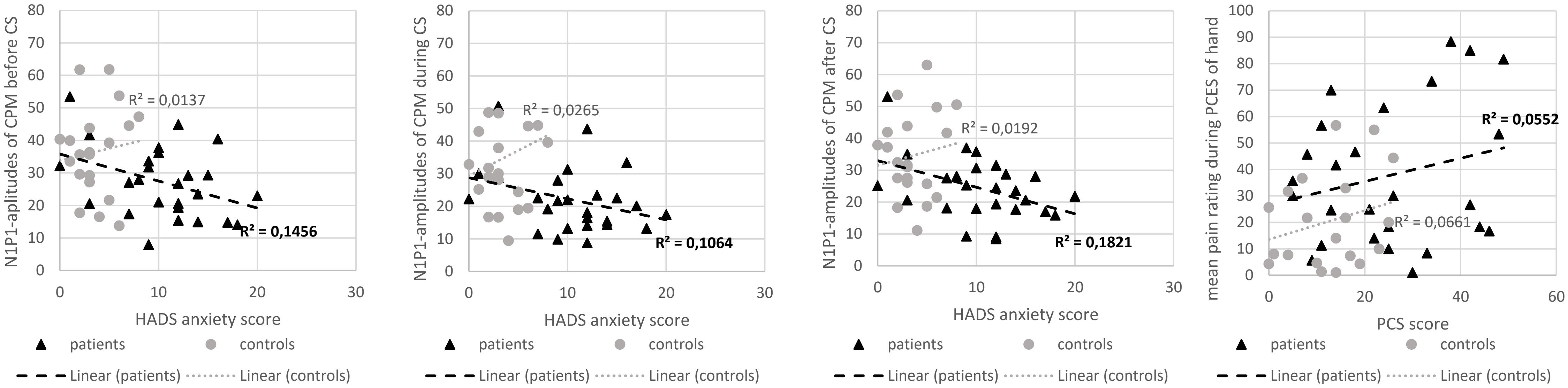

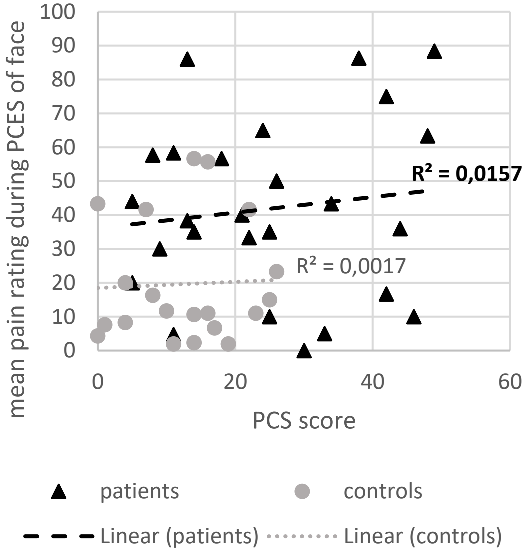

Supplementary figure 1: correlations. DT: detection threshold, PT: pain threshold, PCES: painful cutaneus electrical stimulation, CS: conditioning stimulus, PCS: Pain Catastrophizing Scale, HADS: Hospital Anxiety and Depression Scale, CPM: conditioned pain modulation.
